# Applications of Artificial Intelligence in Temporal Bone Imaging: Advances and Future Challenges

**DOI:** 10.7759/cureus.44591

**Published:** 2023-09-02

**Authors:** Dioni-Pinelopi Petsiou, Anastasios Martinos, Dimitrios Spinos

**Affiliations:** 1 Otolaryngology-Head and Neck Surgery, National and Kapodistrian University of Athens, School of Medicine, Athens, GRC; 2 Otolaryngology-Head and Neck Surgery, Gloucestershire Hospitals NHS Foundation Trust, Gloucester, GBR

**Keywords:** ai and robotics in healthcare, temporal bone imaging, otology, neural networks, machine learning, artificial intelligence

## Abstract

The applications of artificial intelligence (AI) in temporal bone (TB) imaging have gained significant attention in recent years, revolutionizing the field of otolaryngology and radiology. Accurate interpretation of imaging features of TB conditions plays a crucial role in diagnosing and treating a range of ear-related pathologies, including middle and inner ear diseases, otosclerosis, and vestibular schwannomas. According to multiple clinical studies published in the literature, AI-powered algorithms have demonstrated exceptional proficiency in interpreting imaging findings, not only saving time for physicians but also enhancing diagnostic accuracy by reducing human error. Although several challenges remain in routinely relying on AI applications, the collaboration between AI and healthcare professionals holds the key to better patient outcomes and significantly improved patient care. This overview delivers a comprehensive update on the advances of AI in the field of TB imaging, summarizes recent evidence provided by clinical studies, and discusses future insights and challenges in the widespread integration of AI in clinical practice.

## Introduction and background

A series of recent breakthroughs in the evolution of artificial intelligence (AI) has transformed it into a powerful tool in the field of medical diagnostics. The rapid advancements in computational capabilities, coupled with access to massive datasets, have empowered AI to analyze extensive clinical data, including histopathology slides, radiographic images, and other medical imaging modalities [1]. The first AI applications in medicine date back to the 1970s, paving the way for the introduction of personalized medicine in 1999. This milestone has led to significant advancements in prognostic, diagnostic, and therapeutic individualization, shaping the trajectory of medical progress ever since [2,3].

The principles of AI function mainly focus on developing patterns and algorithms that empower machines to learn and make predictions or decisions without being explicitly programmed. In recent years, AI technologies, such as machine learning (ML) and deep learning (DL), have found application in diverse facets of otolaryngology, spanning hearing loss, balance disorders, and investigations into skull base pathology [4]. Given the complexity of these conditions, coupled with the absence of a standardized diagnostic approach, there arises a need for a method that can provide precise interpretation of temporal bone (TB) imaging [5]. While the issuance of diagnostic and treatment guidelines has significantly contributed to this endeavor, their impact on daily clinical practice remains limited. Consequently, a fertile ground has emerged for the integration of AI into clinical settings [4,5]. This study aimed to discuss the latest advancements of AI in TB imaging, as well as to reflect on the challenges in the clinical implementation of ML in the investigation and management of lateral skull base pathology.

## Review

Methods

**Figure 1:**
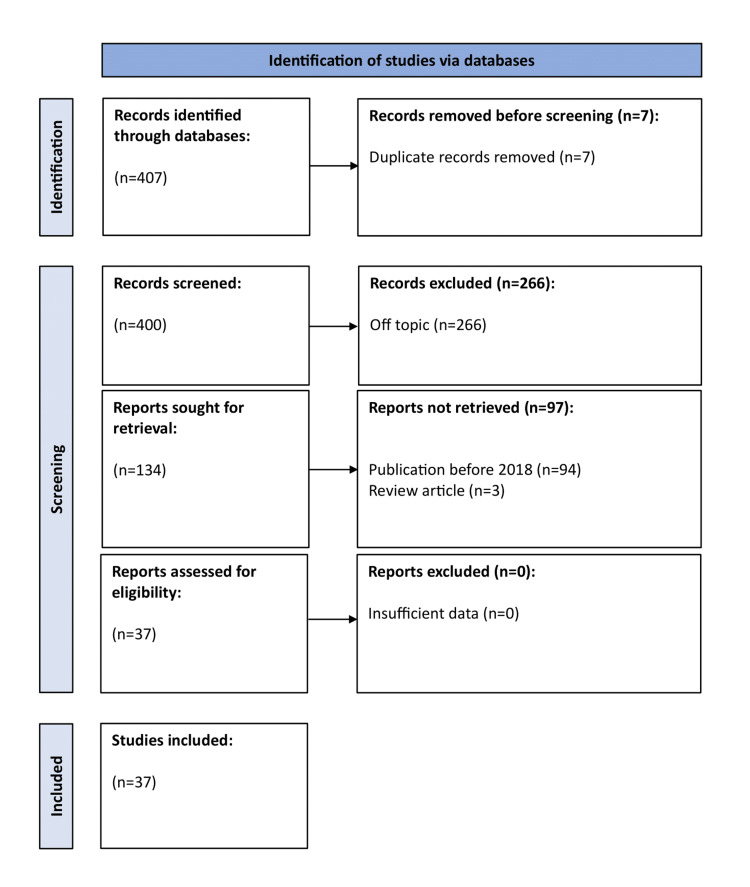
Flowchart of narrative review of the literature.

We conducted a comprehensive search of the literature using bibliographic databases, such as PubMed, Scopus, and Google Scholar. The keywords used included: “artificial intelligence,” “machine learning,” “neural networks,” “temporal bone imaging,” “image segmentation,” “middle ear disease,” “tinnitus,” and “balance disorders.” Our aim was to encompass publications from 2018 onwards. We retrieved and included prospective and retrospective original studies that examined the application of AI in temporal bone imaging for this article. Narrative reviews, systematic reviews/meta-analyses, and studies relevant to the topic but lacking sufficient data were excluded. The article selection strategy involved title and abstract screening, followed by a full-text assessment. At least two authors independently conducted the screening process. Any conflicts were addressed and resolved through mutual consensus among the reviewers. Our research strategy and thought process are reflected in Figure 1. As our article is a narrative review, the article search was not systematically approached and the flowchart does not reflect the Preferred Reporting Items for Systematic Reviews and Meta-Analyses (PRISMA) guidelines [6].

Results and discussion

 *AI Function Principles*

**Figure 2:**
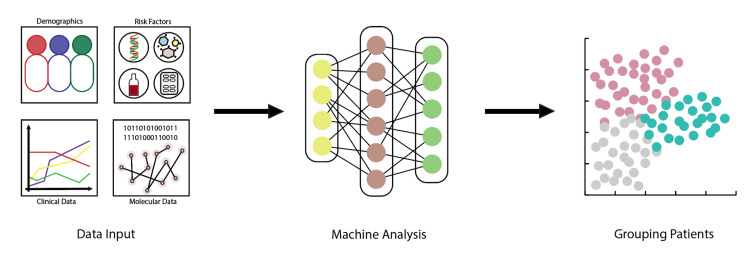
Steps in the machine learning procedure.

The steps of the machine learning (ML) procedure involve data collection, data preprocessing, model training, model evaluation, and model deployment and prediction [7]. These principles of ML function are illustrated in Figure 2. ML can be broadly categorized into four main types: supervised learning, unsupervised learning, semi-supervised learning, and reinforcement learning [7].

Supervised Learning

Supervised learning refers to the use of labeled data in training an ML model. Labeled data consists of input features, such as risk factors for a specific disease or patient demographics, paired with corresponding desired outputs (labels or target variables), such as clinical manifestations or diagnoses [8]. The goal is for the AI model to assimilate the mapping between the input features and the target variables, in order to make accurate predictions or classifications on previously unseen data. Common algorithms used in supervised learning include linear regression, decision trees, random forests, and neural networks [8,9].

Unsupervised Learning

Unsupervised learning addresses unlabeled data without any specific feedback or target variable. The objective of the machine is to discover patterns and correlations within the inputted information, in order to proceed with patient grouping [8]. Unsupervised learning algorithms can perform tasks like clustering, dimensionality reduction, and anomaly detection [8,10]. Clustering allows the formation of natural grouping or clusters within patients of the same dataset without any predefined labels or target variables [11]. Data points within the same cluster are more similar to each other compared to those in different clusters. Dimensionality reduction aims at the simplification and the transformation of high-dimensional data into a lower-dimensional representation, by reducing the number of variables or features and thus preserving only the essential information [12].

Semi-supervised Learning

Semi-supervised learning combines elements of both supervised and unsupervised learning, with the dataset containing a mixture of labeled and unlabeled data [13]. It aims to leverage the smaller amount of labeled data along with a large amount of unlabeled data, in order to improve the learning process [6,14]. By incorporating both information types, semi-supervised learning can effectively address situations where obtaining labeled data is limited, expensive, or time-consuming [15].

Reinforcement Learning

Reinforcement learning describes the trial-and-error training process of the AI agent to maximize a reward signal [16]. Positive or negative feedback engages it in constant interaction with the environment and urges it to opt for the best actions to achieve a cumulative rewarding result over time [17]. Popular reinforcement learning algorithms include Q-Q-learning, deep Q-Q-networks (DQN), and policy gradient methods. Applications in medicine include treatment optimization, healthcare resource allocation, and adaptive medical device development [18].

Deep learning (DL) constitutes a multilayer data processing algorithm using neural networks. DL, along with other AI features has manifested substantial progress in medical imaging recognition tasks [19]. The application of AI in daily clinical practice promises to relieve clinicians’ overburdened work schedules, by minimizing their manual input while facilitating their decision-making process [20,21]. AI applications in radiology are predominantly based on either supervised or unsupervised learning, with the latter having received an increased amount of attention over recent years [20].

AI in temporal bone automated image segmentation

TB imaging, using computed tomography (CT) or magnetic resonance imaging (MRI) scans, plays a crucial role in providing otologists with a holistic perception of the patient’s unique anatomical features. Due to the intrinsic complexity of TB anatomy and its variations, identification of underlying pathology and pre-operative planning is often challenging [22]. Three-dimensional (3D) imaging techniques have been introduced as an addition to the widely used two-dimensional depictions (2D), in order to facilitate the mapping and analysis of the respective anatomy [22].

Apart from 3D reconstructions from 2D image slices, additional technologies that serve different purposes in the field of TB imaging are also in use. Convolutional neural networks (CNN) are classes of artificial deep neural networks applied to the analysis of visual imaging. CNNs are employed to detect and classify specific lesions, like acoustic neuromas or cholesteatomas. The AI system can identify subtle abnormalities and flag potentially concerning areas for further evaluation by healthcare professionals [23]. In terms of diagnosis, computer-aided diagnosis (CAD) systems encompass a wider range of technologies and approaches beyond CNNs. They are software systems designed to assist in the process of medical diagnosis, mainly by differentiating between various ear conditions [22]. Radiomics and texture analysis techniques are responsible for characterizing tissue properties and abnormal changes by extracting quantitative features from image slices. This aids in early disease detection and monitoring treatment responses. In the operative room, the surgeon's field of view may be enriched by AI-powered augmented reality systems, which use radiological images to extract data and overlay critical information. The next link in the chain, medical reports, can benefit from natural language processing (NLP) algorithms, which provide concise summaries for clinicians to review, thus assisting in the documentation process [20].

The most effective assessment method of an AI model’s performance is cross-validation - a dataset fold is chosen as the validation set and the remaining as the training set. The process is repeated multiple times, each time using a different fold as the validation set [24]. Another technique is holdout validation, where the dataset is split into two parts; the training set and the test set. The latter is serving as a proxy for new, unseen data that the model will encounter in real-world conditions [25]. Dice similarity coefficients (DCSs) and Hausdorff distances constitute some of the more commonly used CNN efficiency-metric scales to juxtapose it with the traditional, manual methods [26]. The closer the DSCs and Hausdorff distances are to 1 and 0 mm, respectively, the more similar the AI-performed segmentation to the manual technique [27].

U-Net

U-Net is currently the most popular CNN for precise pixel-level segmentation. The name stems from the shape of the network’s architecture resembling the letter "U" when visualized graphically. It consists of an encoder path and a corresponding decoder path, with skip connections in between [28]. Its ability to produce optimal results while handling limited training data has earned its place as the method of choice in multiple clinical studies [29]. Vaidyanathan et al. used a training dataset of 944 MRI volumes and a validation set of 99 MRI volumes [30]. They aimed to evaluate the 3D U-Net and apply it as a deep-learning tool for inner ear anatomical segmentation. The test Dice similarity coefficient (DSC) scores for the validation cohort vs. the test cohort were 0.86 and 0.82, respectively; the true positive rates were 97.7 and 91.50 and the false discovery rates were 21.8 and 14.8. Among the limitations of the study, the lack of an optimal manual segmentation method was noted, as well as the inability of the model to generalize 5 out of 177 cases, despite the highly marked DCS scores. Overall, it was concluded that the 3D U-Net model is equivalent to the manual technique and it is a dependable and effective approach for inner ear segmentation [30]. Wu et al. suggested a novel 3D U-Net model for the automated segmentation of the semicircular canal, in an attempt to empower a better understanding of the vestibular anatomy [31]. Thirty-nine CT scans were collected and manually annotated by highly skilled physicians. A satisfactory result was noted in most samples, reaching a Dice coefficient (DC) greater than 90% (mean DC: 92.5%) [31]. A study conducted by Heutink et al. used 123 high-resolution CT volumes for automatic segmentation and pre-operative measurement of the cochlea for customized implant planning [32]. Compared to manual annotation, DC was 0.90±0.03 and the average Hausdorff distance was 0.32±0.07 [32]. Hussain et al. trained a 2D U-Net model for inner ear segmentation in micro-CT volumes and found a DC of 0.90 and a Hausdorff distance of 0.74 mm [33].

Materialise Mimics

Materialise Mimics (Leuven, Belgium: Materialise NV) is a medical engineering software with key features, such as 3D image segmentation, 3D model reconstruction, and virtual surgical planning. A recent study by Ke et al. in 2023, applied Mimics software for the delineation and reconstruction of the used structures [22]. They included 80 CT scans interpreting temporal bone structures, 40 in adults and 40 in children. A further sample of 60 annotated CT scans was appointed as the training set. In most structures, no statistically significant difference was identified between the two age groups. The adult and the pediatric set demonstrated a range of DC values from 0.714 to 0.912 and 0.658 to 0.915, respectively. The average symmetric surface distance (ASSD) was less than 0.24 and 0.18 mm for 11 structures in the adult and children groups, respectively. Overall, the segmentation performance was rated as satisfactory and reliable [22].

Other Software

A non-exhaustive list of different software for 3D image segmentation includes the following: AH-Net, ResNet, YOLACT, W‐Net, and 3D cGANs. Neves et al. used a dataset of 24 post-CT and 252 pre-CT volumes and performed a comparison between following three different CNN models: AH-Net, U-Net, and ResNet [23]. The DC for AH-Net was 0.91, 0.85, and 0.75 for inner ear structures, facial nerve, and ossicles, respectively. The average Hausdorff distance was 0.25, 0.21, 0.24, and 0.45 mm. Notably, the study achieved a ninetyfold reduction in CT scan segmentation time, thus highlighting the potential of automated models in relieving physicians from time-consuming procedures. However, the imaging identification does not highlight the importance of distinguishing different anatomical regions and surgical instruments in real-time, while operating [23]. The study by Choi et al. used YOLACT system for real-time segmentation intra-operatively [34]. A total of 5,319 frames from 70 mastoidectomy videos were collected. An accuracy rate of 91.2% and 56.5% was achieved in detecting surgical tools and anatomic regions, respectively. DSC was 48.2% for anatomical segmentation and average frames per second were 32.3. The results were estimated as gratifying, with strong development prospects [34]. Table 1 provides a summary of original studies describing AI applications on automated TB image segmentation.

**Table 1:** Studies describing the use of AI in temporal bone image segmentation. TB: temporal bone; Net: network; CNN: convolutional neural networks; DC: Dice coefficient; ASSD: average symmetric surface distance; AH-Net: anisotropic hybrid network; ResNet: residual neural network; DSC: Dice similarity coefficient; PWD: patch-wise densely connected; YOLACT: You Only Look At CoefficienTs; DSD: deep supervised densely; MSSIM: mean structural similarity index; IoU: intersection over union; PSO: particle swarm optimization; BF: Bayes factors; N/S: not specified

Studies	Study description	Dataset	AI type and/or software	Validation method		Data augmentation	Manual methods used	Key outcomes
Ke et al. 2023 [22]	Automatic segmentation of temporal bone anatomy in adult and pediatric CT scans	80 CT volumes	CNN/Mimics	Cross-validation	Yes		Yes	Adult: DC 0.714-0.912, ASSD <0.24 mm, pediatric: DC 0.658-0.915, ASSD <0.18 mm
Margeta et al. 2022 [35]	A web-based automated image processing research platform in pre-operative temporal bone CT images, combining deep learning and Bayesian inference approaches	60 subjects	CNN/Nautilus	Cross-validation	Yes		Yes	Nautilus demonstrates segmentation performances in the range of previously presented academic results
Neves et al. 2021 [23]	Automated segmentation of temporal bone CT images using CNN	150 CT volumes	CNN/AH-Net, U-Net, ResNet	Cross-validation	Yes		Yes	DC: inner ear 0.91; ossicles 0.85; facial nerve 0.75; sigmoid sinus 0.86. Average Hausdorff distance: 0.25, 0.21, 0.24 and 0.45 mm, respectively
Lv et al. 2021 [36]	Multi-objective segmentation of temporal bone CT images (including the cochlear labyrinth, ossicular chain, and facial nerve) using CNN	30 CT volumes	CNN/W‐Net	Cross-validation	Yes		Yes	DSC: 0.90, 0.85, and 0.77 for the cochlear labyrinth, ossicular chain, and facial nerve, respectively
Wang J et al. 2021 [37]	Proposing a deep learning model for automated segmentation of critical structures in temporal bone CT scans	39 CT volumes	CNN/W-Net	Cross-validation	Yes		Yes	DC and ASSD mean values: normal group -0.703 and 0.250 mm facial nerve; 0.910 and 0.081 mm for labyrinth; 0.855 and 0.107 mm for ossicles, abnormal group -0.506 and 1.049 mm for malformed facial nerve; 0.775 and 0.298 mm for deformed labyrinth; 0.698 and 1.385 mm for aberrant ossicles, respectively
Vaidynathan et al. 2021 [30]	Fully automated segmentation of inner ear on MRI using deep learning	1121 MRI volumes	CNN/3D U-Net	Holdout	Yes		Yes	Mean DSC: 0.8790, true positive rate: 91.5%, false discovery, and false negative rates: 14.8% and 8.49%, respectively
Nikan et al. 2021 [38]	PWD-3DNet (deep learning) for fully automated segmentation of multiple temporal bone structures on CT scans	39 cadaveric TB speciments	CNN/PWD-3DNet	Multiple	Yes		No	DS and Hausdorff distance: average 86% and 0.755 mm, respectively
Hussain et al. 2021 [33]	Auto-context CNN for automatic segmentation of inner ear on CT-scan	17 micro-CT volumes	CNN/2D U-Net + 3D component	Cross-validation	No		Yes	DC 0.90, Hausdorff distance 0.74 mm
Choi et al. 2021 [34]	CNN for video recognition and anatomic detections/segmentation in simple mastoidectomy	5,319 extracted frames	CNN/YOLACT	Holdout	No		Yes	Mean detection accuracies of surgical tools and anatomic regions: 91.2% and 56.5%, respectively; mean DSC 48.2%; mean frames/second 32.3
Wu et al. 2021 [31]	A3D U-Net with attention mechanism for automatic semicircular canal segmentation of CT scans	39 CT volumes	CNN/3D U-Net	Holdout	N/S		Yes	Mean DC 92.5%
Ahmadi et al. 2021 [39]	Combination of micro-CT/MRI developments and modern neuroimaging technology: development of a novel in-vivo atlas and template of the human inner ear	MRI from 63 subjects	Modern neuroimaging technology	Holdout	Yes		Yes	Publishing a comprehensive list of inner ear landmarks for distance measurements
Jeevakala et al. 2020 [40]	Automatic method for internal auditory canal and nerves detection and segmentation	50 patients	CNN/Mask R-CNN, ResNet-50 model	Holdout	No		Yes	Mean IoU of ResNet-50 and ResNet-101: 0.79 and 0.74, respectively; DS using region growing, PSO and U-Net method: 92%, 94%, and 96%, respectively
Li et al. 2020 [41]	3D Deep Supervised Densely Network for temporal bone segmentation of CT scans	64 CT volumes	CNN/3D-DSD Net	Holdout	Yes		Yes	Average DSC: 77.18%, average ASD: 0.20 mm, and average AVD: 0.43 mm
Heutink et al. 2020 [32]	Multi-scale deep learning for cochlea localization, segmentation, and analysis on CT scans	123 CT volumes	CNN/U- Net-like	Holdout	Yes		Yes	Average DC 0.90, BF score of 0.95, average Hausdorff distance 3.05 and 0.32 against manual method
Wang et al. 2019 [42]	3D generative adversarial nets for metal artifact reduction for cochlea segmentation in CT images	24 post-CT and 252 pre-CT volumes	CNN	Holdout	Yes		No	3D superior to MSSIM; 3D architecture superior to 2D

AI in middle ear disease

Chronic Otitis Media With or Without Cholesteatoma

AI’s role in the management of chronic otitis media (COM) has been well-established, including image analysis, automated diagnosis, surgical planning, treatment recommendations, monitoring, and prognostication [43,44]. Multiple software programs, including CNN, VGG-16, and MobileNetV2, have been used for the detection of COM [45-58]. Studies involving AI technologies in middle ear diseases are presented in Table 2.

**Table 2:** Studies involving AI technologies in middle ear disease. HRCT: high-resolution CT; Net: network; CNN: convolutional neural networks; LNN: logical neural network; TM: tympanic membrane; AOM: acute otitis media; OM: otitis media; COM: chronic otitis media; OME: otitis media with effusion; ResNet: residual neural network; VGG: Visual Geometry Group; NASNet: neural architecture search network; AUC: area under the curve; N/S: not specified

Studies	Study description	Dataset	AI type and/or software	Validation method		Data augmentation	Manual methods used	Key outcomes
Tseng et al. 2023 [45]	CNN for diagnosis of cholesteatoma	834 otoscopic images	CNN/DenseNet201, NASNetLarge, MobileNet-v2	Holdout	Yes		Yes	Accuracies for differentiating cholesteatoma from - normal 83.8-98.5%; abnormal non-cholesteatoma 75.6-90.1%; non-cholesteatoma 87.0-90.4%
Ayral et al. 2023 [46]	AI in differential diagnosis of chronic otitis media with and without cholesteatoma	300 CT images	CNN/ResNet-50, MobileNet-v2	Holdout	No		No	Overall accuracy rate: 93.33% ResNet-50; 86.67% MobilNet-v2. Diagnostic accuracy rates: ≥90% ResNet-50; ≥80% MobileNet-v2
Hasan et al. 2023 [47]	A computer vision algorithm for classification of mastoid process pneumatization on temporal bone CT scans	784 CT images	CNN	Holdout	Yes		Yes	Overall accuracy 0.954, sensitivity 0.860, specificity 0.989, positive predictive value 0.973, negative predictive value 0.935, false positive rate 0.006
Takahashi et al. 2022 [48]	AI in pre-operative prediction for mastoid extension in pars flaccida cholesteatoma using high-resolution CT scans	164 patients	CNN/MobileNet-v2	Cross-validation	Yes		Yes	Average accuracy of ensemble prediction model 81.14% (sensitivity 84.95%, specificity 77.33%) vs. manual 73.41% (sensitivity 83.17%; specificity 64.13%)
Eroğlu et al. 2022 [49]	AI in differential diagnosis of chronic otitis media with and without cholesteatoma	200 patients	CNN/AlexNet, GoogLeNet, DenseNet-201	Holdout	No		Yes	Accuracy rate 95.4% (correctly predicted 2952 out of 3093 CT images, 141 incorrectly predicted)
Chen et al. 2022 [50]	Smartphone-based AI for detection and diagnosis of middle ear diseases	2820 eardrum images	CNN/VGG16, VGG19, Xception, Inception-v3, NASNetLarge, ResNet-50	Holdout	Yes		Yes	Detection accuracy for binary outcomes: 98%, recognition accuracy: 97.6% vs. detection accuracy from general physicians, resident doctors, and otolaryngology specialists: 36%, 80%, 90%, respectively
Duan et al. 2022 [51]	AI in diagnosis of temporal bone diseases, including cholesteatoma and Langerhans cell histiocytosis	119 patients	CNN/VGG16_BN	Holdout	Yes		Yes	Physician vs. AI: accuracy (cholesteatoma) 0.99 vs. 0.89, (Langerhans cell histiocytosis) 0.99 vs. 0.97, (middle ear inflammation) 0.99 vs. 0.89
Wang et al. 2022 [52]	Deep-learning method for the diagnosis of different chronic middle ear diseases, including middle ear cholesteatoma and chronic suppurative otitis media	973 ears	CNN/Mask R-CNN, VGG-16	Cross-validation	Yes		Yes	Average precision 90.1%, recall 85.4%, F1 score 87.2%
Byun et al. 2022 [53]	Assesses the performance of the teachable machine for TM lesion diagnosis	3024 TM images	Machine learning/teachable machine	Holdout	Yes		No	Overall accuracy of the classification of the 80 representative tympanic membrane images: 78.75%, hit rates for normal, OME, COM, and cholesteatoma: 95.0%, 70.0%, 90.0%, 60.0%, respectively
Tan et al. 2021 [54]	Analyzes the clinical performance of otolaryngologists in diagnosing fenestral otosclerosis (OS) and develops a deep learning model for OS diagnosis	134,574 CT slices	LNN	Holdout	Yes		Yes	Area under the curve (AUC): 99.5%
Wang et al. 2020 [55]	Deep learning in diagnosis of COM: CT scan-based	672 CT images	CNN/Inception-v2 (Mountain View, CA: Google LLC)	Cross-validation	Yes		Yes	Physicians vs. deep learning: sensitivity 83.3% vs. 81.1, specificity: 91.4% vs. 88.8
Khan et al. 2020 [56]	CNN in detection of tympanic membrane and middle ear infection from oto-endoscopic images	2484 otoendoscopic images	CNN/ResNet, VGGNet, GoogLeNet, DenseNet,	Multiple	Yes		Yes	Physicians average accuracy: 74% vs. AI: 87%
Tran et al. 2018 [57]	AI for diagnosis of pediatric OM	1230 otoscopic images	Automatic algorithm	Cross-validation	No		Yes	Max classification accuracy 91.41% (OME vs. AOM)
Fujima et al. 2021 [58]	AI for interpretation of temporal bone CT images in patients with otosclerosis	198 CT images	AlexNet, VGGNet, GoogLeNet, ResNet	Holdout	Yes		Yes	Diagnostic accuracies: 0.89, 0.72, 0.81, 0.86, and 0.86 for the radiologist, AlexNet, VGGNet, GoogLeNet, and ResNet, respectively

In 2022, Eroğlu et al. conducted a three-group study with 100 participants in each [49]. The first group consisted of patients who had chronic otitis media with cholesteatoma (CHO), the second group consisted of patients with chronic otitis media without cholesteatoma (COM), and the third group consisted of participants without disease (control group). The CHO group underwent tympanoplasty with or without further mastoid exploration. Four physicians blindly created a homogenous dataset with 8-10 CT images of each participant. Results were initially retrieved using three pre-trained architectures, AlexNet, GoogLeNet, and DenseNet-201. After presenting the input images to the deep neural network, feature maps were created, leading to data classification as the final step. The performance of the software was evaluated using holdout validation, splitting the group of images into two parts, the training and the testing set. The highest accuracy rate was reported in AlexNet (99.44% in the CHO group), followed by DenseNet-201 (91.76%) and GoogLeNet (84.65%). These outcomes supported that deep learning networks can considerably assist physicians by increasing their diagnostic effectiveness and contributing to the improvement of the treatment course and outcomes of each patient. The limitation of this study was the small sample of participants [49]. A similarly designed study was published by Ayral et al. in 2023, assessing the effectiveness of ResNet-50 and MobileNet-v2 models [46]. The two architectures achieved an overall accuracy rate of 93.3% and 86.7%, respectively. The diagnostic accuracy rates for the ResNet-50 and MobileNet-v2 models were 100% and 95% for the CHO patients, 90% and 85% for the COM patients, and 90% and 80% for controls, respectively [46].

Wang et al. described a deep learning model called “Middle Ear Structure Identification Classifier” (MESIC) [52]. This technology was aimed to facilitate the diagnosis of chronic middle ear conditions, including chronic suppurative otitis media and cholesteatoma disease. The study used a dataset of 973 ears, created by an otolaryngologist. Each CT scan was manually labeled as middle ear cholesteatoma (MEC), chronic suppurative otitis media (CSOM), or normal. Mask R-CNN was used to automatically interpret CT scan findings. Data classification was mainly performed by VGG-16. The network’s performance was validated using average precision, recall, and F1-score. Results were 90.1%, 85.4%, and 87.2% for each of the three factors, respectively. Thus, it was demonstrated that MESIC is a cost-effective and efficient means to identify and differentiate between CSOM and MEC [52].

Otosclerosis

Neural networks and particularly deep learning models, seem to be valuable tools in the early diagnosis of fenestral otosclerosis. AI networks allow clinicians to make early diagnoses of otosclerosis, by differentiating its characteristic features in imaging from normal anatomical structures (e.g., abnormal bony thickening and sclerosis around the oval window) [59].

Fujima et al. in 2021 were the first to use different deep-learning models to interpret temporal bone CT images of individuals with otosclerosis [58]. AlexNet, VGGNet, GoogLeNet, and ResNet were the architectures used. One hundred and ninety-eight CT images were interpreted both by the AI models and a trained radiologist. The architectures’ performance was evaluated via the holdout method, with the training set comprising 140 CT scans and the test set of 58. The diagnostic accuracies for the radiologist, AlexNet, VGGNet, GoogLeNet, and ResNet were 0.89, 0.72, 0.81, 0.86, and 0.86, respectively. As a result, the study failed to demonstrate a significant inferiority of the AI models in comparison to the radiologist’s performance [58]. In 2021, Tan et al. examined the application of LNN in the diagnosis of fenestral otosclerosis (OS) using temporal bone HRCT scans [54]. A total of 31,744 CT slices obtained from 144 patients were used as the neural network’s test set. The VGG-19 software served as the backbone of the neural network model. Adam optimizer was used to reduce the bounding box refinement of the LNN model and prevent classification losses. The LNN performance was subsequently compared to the diagnoses reported by seven physicians. The study showed that the sensitivity (96.4%) and specificity (98.9%) presented by the LNN model exceeded the sensitivity and specificity of the physicians [54].

Langerhans Cell Histiocytosis

AI’s capabilities in medical image analysis and pattern recognition can be leveraged into a widened spectrum of diseases, including rarer entities, such as Langerhans cell histiocytosis (LCH). The incidence of LCH varies from one or two per million in adults and one to eight per million in children. Otologic manifestations are present in about 40% of LCH patients [60,61].

Possible AI applications in TB LCH diagnosis were discussed by Duan et al. in 2022 [51]. In the patient dataset comprising a total of 119 patients, TB LCH was histologically proven in 41 individuals. CT images were classified by the VGG16_BN neural network model, an architecture with approximately 138 million network parameters. The network uses a convolutional layer, two interconnected layers, and a soft maximum output. The efficiency of VGG16_BN was ensured by applying various image processing techniques. The model’s performance was subsequently compared to a clinician’s methods. The results reported a receiving operating characteristic of 0.99 vs. 0.98, accuracy of 0.99 vs. 0.97, and specificity of 0.99 vs. 0.97. LCH is often difficult to assess and reaching a diagnosis can be delayed due to its rarity. Approaching TB LCH with the contribution of artificial networks is a very important step in improving the clinical outcomes of patients with this rare condition [51].

AI in tinnitus and balance disorders

Intriguing novel approaches in classifying imaging findings for tinnitus and vertigo patients have been described in the literature. Table 3 summarizes key studies describing AI applications on patients with tinnitus and/or balance disorders. Among the main targets of interest are Meniere’s disease and benign paroxysmal positional vertigo.

**Table 3:** Studies covering AI applications in tinnitus and balance disorders. CNN: convolutional neural networks; BERT: bidirectional encoder representations from transformer; AUC: area under the curve; MLP: multi-layer perception network; LSTM: long short-term memory model; INHEARIT: inner-ear hydrops estimation via artificial intelligence; N/S: not specified

Studies	Study description	Dataset	AI type and/or software	Validation method		Data augmentation	Manual methods used	Key outcomes
Li et al. 2022 [62]	Novel approach in classifying actionable radiology reports of tinnitus patients	5864 CT reports	CNN, MLP, Bi-LSTM, hybrid Bi-LSTM-CNN	Holdout	No		Yes	BERT AUC-0.868, F1-0.760 compared with that of the Word2vec-based models AUC-0.767, F1-0.733 on validation data
Park et al. 2021 [63]	Deep learning in measuring endolymphatic hydrops ratios in MRIs of patients with Ménière disease	MRI of 124 subjects	Neural networks/3into3 Inception, 3into U-Net	Cross-validation	Yes		Yes	Physicians vs. INHEARIT-v2 system: average intraclass correlation coefficient for all cases 0.941; average intraclass correlation coefficient of the vestibules 0.968, and that of the cochleae 0.914

The study of Li et al. aimed to propose the use of a novel tool in actionable radiology reports classification in individuals with tinnitus [62]. This approach uses bidirectional encoder representations derived from BERT-based software. The interpretation of 5864 CT scans was initially conducted by two radiologists and then compared to a deep-learning neural network’s performance. In comparison to the Word2vec-based models, the BERT-based model showed a superior result (AUC: 0.868, F1: 0.760) [62].

Investigating a novel tool in the diagnostics of Meniere’s disease, Park et al. proposed the use of 3into3 Inception and 3into U-Net networks to analyze endolymphatic hydrops (EH) ratios via MRI [63]. The two models were integrated into the newly developed INHEARIT-v2 architecture. The study enrolled 124 participants. The performance values for the 3into Inception and 3into U-Net networks were 0.743 and 0.811, respectively. Comparing the results of the trained physicians to the INHEARIT-v2 performance, a high correlation was found between the EH ratio values measured by the automated system and the experts [63].

AI in vestibular schwannoma

Currently, the diagnosis, stratification of radiotherapy dosage, and follow-up measurements of vestibular schwannoma require delineation by manually reviewing MRI images [64]. Even though this process can be successfully undertaken by well-trained experts, it is undeniably tedious and time-consuming. As a result, there is a considerable effort underway to introduce AI learning algorithms in order to automate tumor contouring. Table 4 includes original studies applying AI software to the diagnosis and clinical management of vestibular schwannoma.

**Table 4:** Studies applying AI software to the diagnosis and clinical management of vestibular schwannoma. VS: vestibular schwannoma; ANN: artificial neural network; AUC: area under the curve; MR: magnetic resonance; MRI: magnetic resonance imaging; CNN: convolutional neural network; DC: dice coefficient; S2S: surface-to-surface; BM: brain metastasis; GKRS: Gamma Knife radiosurgery; RVD: related volume difference; N/S: not specified

Studies	Study description	Dataset	AI type and/or software	Validation method		Data augmentation	Manual methods used	Key outcomes
Abouzari et al. 2020 [65]	Use of artificial neural network to predict vestibular schwannoma recurrence	789 VS patients	ANN	Holdout	No		No	Superior performance of ANN compared to the regression model (AUC: 0.79; p: 0.001). Higher sensitivity (61%) and specificity (81%). Correctly classified 70% of cases
Shapey et al. 2021 [66]	Segmentation of MRI images in vestibular schwannoma patients through an open annotated dataset and baseline algorithm	484 MR images	CNN/2.5 D U-Net	Holdout	No		Yes	Average DC of 94.5% for T1 images and 90.7% for T2 images
Lee et al. 2020 [67]	CNN in analyzing multi-parametric MR images of VS patients	516 patients	CNN/2-pathway 3D U-Net	Cross-validation	No		Yes	Mean DC of 0.90±0.05 for the 2-pathway model VS 0.87±0.07 for the single-pathway model
Lee et al. 2021 [68]	AI applications in longitudinal imaging analysis of vestibular schwannoma following radiosurgery	861 VS patients (1290 MR examinations)	CNN/U-Net (dual pathway and single pathway model)	Cross-validation	No		Yes	RVD between AI and manual measurements: +1.74%, -0.31%, - 0.44%, -0.19%, -0.01%, and +0.26% at each follow‐up point
Neve et al. 2022 [69]	Fully automated 3D vestibular schwannoma segmentation with and without gadolinium-based contrast material	MRIs from 214 patients	CNN/3D U-Net	Cross-validation	No		Yes	Mean S2S distance of less than 0.6 mm for the T1-weighted model. T2-weighted images with a mean S2S distance of less than 0.6 mm. The tool was similar to human delineations in 85-92% of cases
Lee et al. 2023 [70]	Lesion delineation framework for vestibular schwannoma, meningioma, and brain metastasis for gamma knife radiosurgery using stereotactic magnetic resonance images	506 VS patients, 1,069 meningioma patients, 574 BM patients with BM who had been treated using GKRS	CNN/3 D U-Net	Holdout	No		Yes	DC of 0.91±0.05 vs. 0.90±0.06, and 0.82±0.23 vs. 0.78±0.34 (2 parametric vs. single parametric model for VS and BM respectively). DC of 0.83±0.17 vs. 0.84±0.22 (for meningioma, respectively).

The study of Lee et al. aimed to develop an algorithm to automate imaging analysis of vestibular schwannoma following radiosurgery [68]. An end-to-end deep-learning scheme with an automated pre-processing pipeline was developed and consequently applied to a series of 1290 MR examinations that included T1-weighted contrast-enhanced (T1WC) and T2-weighted (T2W) parametric magnetic resonance (MR) images. The images were derived from a sample of 861 consecutive patients who underwent Gamma Knife radiosurgery (GKRS) between 1993 and 2008. The AI measurements were then compared to the clinical measurements done manually by expert radiologists. The relative volume difference (RVD) between the former and latter was +1.74%, -0.31%, -0.44%, -0.19%, -0.01%, and +0.26% at subsequent follow-up points. The performance of the models was evaluated using the Dice coefficient. The study concluded that the proposed AI model could be applied in the follow-up of Gamma knife radiosurgery for vestibular schwannoma [68].

Yang et al. utilized a two-level machine-learning model to predict the long-term outcome and transient pseudo-progression after GKRS [71]. Three hundred thirty-six patients were included in the study. The evaluation of long-term outcomes was based on five radiomic features describing the variation of T2W intensity and inhomogeneity of contrast enhancement in the tumor. The prediction of long-term outcomes achieved an accuracy of 88.4%. The prediction of transient pseudoprogression, based on another five radiomic features associated with the inhomogeneous hypointensity pattern of contrast enhancement and the variation of T2W intensity, achieved an accuracy of 85.0% [71].

In 2021, Shapey et al. described the use of a previously developed novel AI framework based on a 2.5D CNN with the aim to utilize the difference between in-plane and through-plane resolutions encountered in typical imaging protocols [66]. The automatic segmentation results were compared to the results of manual segmentations using the DS, average symmetric surface distance (ASSD), and relative volume error (RVE), achieving excellent results. The dataset consisted of 484 MR images collected from 242 consecutive patients undergoing Gamma Knife stereotactic radiosurgery (GKSR). The dataset included segmentations and contours used in treatment planning, dose details, and co-registration assessed by radiologists. Compared to the manual methods, the CNN yielded average DCS of 99.9±0.2% for T1 images and 97.6±2.2% for T2 images, with all DCS higher than 88%. An automatic segmentation algorithm trained on the dataset demonstrated high agreement with an average DS of 94.5±2.2% for T1 images and 90.7±3.6% for T2 images, comparable to inter-observer variability between clinical annotators (average DS of 93.82±3.08%). The study showcased the superiority of the 2.5D U-Net implementation over other baseline neural networks, yielding improvements in DS of more than 3% [66].

Another CNN application on automated vestibular schwannoma segmentation using contrast-enhanced T1- and T2-weighted MRI scans was presented by Neve et al. in 2022 [69]. The CNN achieved a mean surface-to-surface (S2S) distance of less than 0.6 mm for both whole tumor and intrameatal/extrameatal tumor parts in the independent test sets. The Dice index and Hausdorff distance were reported as 0.92 and 2.1 mm, respectively, for T1-weighted images, and 0.87 and 1.5 mm, respectively, for T2-weighted images in the independent test set. The observer study indicated a similarity between the automated tool and human delineations in 85-92% of cases. This automated segmentation method may hold the potential to aid clinical diagnosis and treatment planning in vestibular schwannoma cases [69].

Lee et al. in 2023 focused on target delineation in GKRS for smaller intra-cranial tumors using deep learning-based algorithms [70]. Stereotactic MR images from 506 patients with vestibular schwannoma (VS), 1,069 patients with meningioma, and 574 patients with brain metastases (BM) undergoing GKRS were collected. The developed algorithm utilized a three-dimensional patching-based training strategy and dual-pathway architecture to handle inconsistent field-of-views and anisotropic voxel sizes. For VS and BM, the model trained using two-parametric MR images outperformed the model trained using single-parametric images, showing median Dice coefficients of 0.91 (two-parametric) vs. 0.90 (single-parametric) for VS and 0.82 (two-parametric) vs. 0.78 (single-parametric) for BM. For meningioma, the dual-pathway model was dominated by single-parametric images, achieving median Dice coefficients of 0.83 (dual-pathway) vs. 0.84 (single-parametric). Combining three data sets for training led to comparable or even higher testing median DCS for all three diseases using two-parametric input: VS (0.91), meningioma (0.83), and BM (0.84). The proposed deep learning-based segmentation scheme demonstrated successful application in intra-cranial tumor segmentation for GKRS planning, effectively leveraging stereotactic MR image volumes [70].

In 2020, Lee et al. compared the manual delineation of VS imaging to a deep learning method utilizing a two-pathway U-Net model involving different convolution kernel sizes to extract in-plane and through-plane features of anisotropic MR images [67]. The dataset consisted of multi-parametric MR images collected from 516 VS patients. Additionally, the researchers used multi-parametric MR images with different contrasts for training to effectively segment tumors with both solid and cystic parts. The automatic segmentation results showed that the two-pathway model outperformed the single-pathway model in terms of DS (0.90±0.05 vs. 0.87±0.07) using T1W, T1WC, and T2W anisotropic MR images. Furthermore, the two-pathway models trained using bi-parametric (T1WC and T2W) and tri-parametric (T1W, T2W, and T1WC) images showed improved segmentation of non-homogeneous tumor parts compared to the model trained using single-parametric (T1WC) images, with Dice scores of 0.89±0.05 and 0.90±0.05, respectively, larger than 0.88±0.06. The proposed two-pathway U-Net model proved to be superior to the single-pathway model for VS segmentation using anisotropic MR images, while the multi-parametric models effectively enhanced segmentation by distinguishing between solid and cystic tumor components [67].

Limitations and ethical dilemmas

Despite the promising advancements in AI applications for TB imaging, there are notable limitations and ethical considerations that warrant careful attention. Acknowledging these areas of struggle is important for a balanced and responsible deployment of AI in interpreting TB imaging.

First of all, AI algorithms require large and diverse datasets to learn effectively. In the case of TB imaging, if the dataset is small, obtaining high-quality annotated images may be challenging, leading to potential biases or reduced performance. Limited exposure to training data might also complicate the identification of uncommon anatomical variations or unusual pathologies. The high quality of the training datasets is also very important to maintain, as it will prevent the algorithm from being biased. Furthermore, as long as the medical field evolves rapidly, AI systems need mechanisms to continually learn, adapt, and update based on new information [3].

It is also important to ensure the attainment of multiple ethical parameters when applying AI in medical practice. To begin with, sharing medical records and images with AI algorithms raises concerns about data confidentiality and security. Although the formation of large datasets is necessary, there is currently no common system between institutions, that could ensure the safety of the transferred data. Additionally, it is crucial for patients to be informed about how AI is being used in their healthcare and to provide consent for AI-driven diagnostics or treatments. Another ethical consideration touches on issues of accountability and liability, as we are in need of clear guidelines on who will be appointed responsible in case the AI system makes a wrong diagnosis or recommendation. Concerns about equal access to AI-driven applications in healthcare are also raised, as patients from lower socioeconomic backgrounds may not be favored from such expensive or source-intensive treatments. Finally, it is necessary to maintain an effective, fruitful, and healthy collaboration between physicians and AI technologies, in order to ensure that AI does not replace human competency and decision-making [3,4].

Future outlook

As AI technologies continue to advance, their future implications are multi-faceted and extend across various aspects of clinical care. AI helps manage the overwhelming volume of medical data by monitoring large patient datasets. it aids in organizing patient records, streamlining administrative tasks, and improving the overall efficiency of healthcare operations. Furthermore, AI systems are poised to become essential tools for diagnosing medical conditions accurately and rapidly. Future AI models are expected to analyze even more complex medical data, including images, lab results, and patient histories. They can also contribute to personalized treatments by considering factors like genetics and treatment outcomes from similar cases. Applications of AI are also expected to advance in the field of telehealth and remote monitoring. With the help of wearable devices and sensors, telehealth platforms integrated with AI intervene promptly and provide remote consultations, reducing hospital readmissions. Finally, AI offers variable contributions to medical training by enhancing hands-on experiences, improving skill acquisition, and preparing healthcare professionals for real-world scenarios. This can be achieved, among others, by AI-powered simulations and virtual reality platforms, interactive anatomical models, and real-time feedback.

## Conclusions

Compared to previous years, there has been a notable surge in the number of studies incorporating AI algorithms and deep learning architectures in temporal bone imaging. This growing trend indicates the increasing recognition of AI's potential to enhance diagnostic accuracy, improve surgical planning, and optimize patient care in Otolaryngology. However, AI technological advances need to be approached with caution, as its current limitations and challenges cannot be overlooked.

It is also important to note that while AI applications are implemented to assist healthcare professionals, they are not meant to replace human expertise. Instead, AI acts as a supportive tool, providing insights and facilitating the diagnostic procedure. As the technology continues to advance, we can expect AI to further improve and expand its applications, supporting the transformation of traditional medical practices.
